# Tensile Properties of Composite Reinforced with Three-Dimensional Printed Fibers

**DOI:** 10.3390/polym12051089

**Published:** 2020-05-10

**Authors:** Komal Agarwal, Rahul Sahay, Avinash Baji

**Affiliations:** 1 Xtreme Materials Laboratory (XML), Singapore University of Technology and Design, Singapore 487372, Singapore; komal_agarwal@mymail.sutd.edu.sg (K.A.); rahul@sutd.edu.sg (R.S.); 2 Department of Engineering, School of Engineering and Mathematical Sciences (SEMS), La Trobe University, Bundoora 3086, Australia

**Keywords:** melt-electrospinning, composites, fibers, additive manufacturing

## Abstract

This study used melt-electrospinning writing to fabricate three-dimensional fiber constructs by embedding them in a polyvinyl alcohol (PVA) matrix to obtain thin composite films. Fourier transform infrared spectroscopy (FTIR) and dynamic scanning calorimetry (DSC) were used to demonstrate an interaction between the polycaprolactone (PCL) fibrous phase and the PVA matrix phase. Following this, the mechanical deformation behavior of the composite was investigated, and the effect of reinforcement with three-dimensional fibrous constructs was illustrated. The specific strength of the composite was found to be five times higher than the specific strength of the neat PVA matrix. Additionally, the specific toughness of the composite was determined to be roughly four times higher than the specific toughness determined for the neat PVA matrix. These results demonstrate the potential of using melt-electrospinning writing for producing three-dimensional fibrous constructs for composite reinforcement purposes.

## 1. Introduction

Fiber reinforced composites are an emerging class of materials that are increasingly investigated for a wide range of applications, including biomedical, energy, packaging, and advanced structural applications [1,2,3,4,5]. Among all the processing technologies, electrospinning has gained widespread attention as one of the simplest and straightforward fabrication techniques to produce fibers for composite reinforcement [6,7]. The fibers produced using this technique have a large length to diameter ratio and large surface area. The high draw ratio and elongation experienced by the fibers during the electrospinning process aligns the molecular chains along the fiber axis. This explains why electrospun fibers can display enhanced mechanical strength and stiffness compared its bulk counterparts [8,9,10,11]. These attributes of electrospun fibers make them suitable candidates as reinforcements in composites [6]. Besides improving the strength and stiffness of the composites, the electrospun fibers can also be used to improve the thermal, optical, or conductive properties of the composites [12,13,14,15,16,17,18,19,20,21].

Most studies report two common fabrication techniques to produce electrospun fiber reinforced composites: viz. dip-coating and film-stacking methods [6,16,17,22,23,24,25]. The reinforcing effect of the electrospun fibers in these composites is greatly influenced by the thickness of the fiber membranes, dispersion, and arrangement of the fibers within the matrix. Despite the advantages of electrospinning in producing fiber reinforced composites, the use of the conventional electrospinning setup has limited success in producing controlled three-dimensional architectures for composite reinforcement. It is argued that embedding well controlled 3D fibrous architectures within a composite can further improve the mechanical performance of the fiber reinforced composites [15,16,17,18,19,20,21,22]. Recently, some studies have explored the melt-electrospinning setup to deposit fibers in a controllable manner [26,27,28]. In the melt-electrospinning writing setup, the electrospinning voltage and the distance between the electrospinning nozzle and collector are lowered. As the polymer jet ejected from the electrospinning nozzle does not experience whipping instabilities, it ensures that the fibers can be deposited in a precise manner to obtain three-dimensional fiber patterns [27].

The aim of this study is to demonstrate that melt-electrospinning writing, that is commonly used to produce three-dimensional scaffolds for tissue engineering applications, can also be used to produce three-dimensional fibrous structures for composite reinforcement applications. In the first step, the three-dimensional grid patterns were produced using melt-electrospinning writing. The grid patterns produced using melt-electrospinning writing were based on polycaprolactone (PCL) fibers. PCL was chosen in this study due to its low melting point (≈ 60 °C) and ability to produce three-dimensional structures using melt-electrospinning writing. These three-dimensional fiber grid structures were then embedded in polyvinyl alcohol (PVA) matrix, and their mechanical deformation behavior was investigated. PVA was chosen as the matrix material due to its water solubility and ability to form films. Since the fibers were melt extruded out of the nozzle and then deposited on a collector, the surface of the fibers tended to fuse with the fibers on top of which they were deposited. This also ensured that the fibers present at the interconnecting junctions within the grid bind strongly with one another. Embedding these fiber structures within the matrix demonstrated improvement of the mechanical properties of the composite.

## 2. Experimental

### 2.1. Electrospinning Writing

Polycaprolactone (PCL, *M*_w_ = 80,000) was obtained from Sigma Aldrich (North Ryde, NSW, Australia) for electrospinning writing. A commercial electrospinning apparatus (Novaspider, Gipuzkoa, Spain) was used in this study as a 3D printer to obtain PCL fibrous structures. The instrument was controlled and monitored using a web interface (OctoPrint, OctoPrint.org). Melt electrospinning writing was performed at ambient conditions. PCL pellets were fed into an extruder that was mounted on an XYZ translation stage, and its movement was controlled using a computer program written in G-code. The extruder was heated to 120 °C to ensure that the PCL remained in a molten state. PCL was then extruded out through a nozzle with a 0.4 mm diameter. The distance between the collector plate and the nozzle was set at 5 mm, and the voltage applied was 3 kV. The fibers were collected in a grid pattern of 100 mm × 100 mm.

### 2.2. Composite Fabrication

The composites were produced by embedding 90 mg of electrospun PCL in polyvinyl alcohol (PVA, *M*_w_ = 130,000, North Ryde, NSW, Australia). For this purpose, 10 wt % of PVA solution was first prepared by dissolving PVA in water. The solution was then filled in a syringe and sprayed on top of printed PCL fibers. Following this, the samples were then left to dry in a vacuum desiccator for 72 h. This produced PVA reinforced with PCL fibers.

### 2.3. Characterization

The microstructure of the samples was examined using scanning electron microscopy (SEM, JEOL, JSM-6700F, JEOL Asia, Singapore) at an accelerating voltage of 5 kV. The samples were sputter-coated (Joel JFC-1600, JEOL Asia, Singapore) with a thin layer of gold (18 mA, 60 s) before they were examined under SEM.

Differential scanning calorimetry (DSC, DSC Q20 TA Instruments, TA Instruments, Singapore) was used to examine the melting temperature of the samples. The temperature was ramped at 3 °C·min^−1^ from −60 to 250 °C, in a nitrogen atmosphere with a flow rate of 40 mL·min^−1^.

Fourier transform infrared spectroscopy (FTIR) spectra of the samples were collected using a Bruker Alpha spectrometer (Bruker Singapore Pte. Ltd, Singapore). FTIR spectra were recorded at a resolution of 4 cm^−1^, with an average of 256 scans at room temperature.

The specimens for the tensile test were trimmed using computer integrated laser cutting (GCC LaserPro Spirit GLS Hybrid, Uscribe, Wendouree, Victoria, Australia) to avoid pulling or shear deformation of the samples. The samples were laser cut at 40% power control and 2032 mm·s^−1^ motor speed. The ends of the samples were carefully glued to a cardboard sheet using superglue to ensure that the samples were gripped firmly during the tests. The dimensions of the specimens were fixed at 25 mm × 5 mm (L × W). The thickness of the samples were ≈ 120 μm. A 100 N load cell was used during the test, and the samples were pulled at a rate of 5 mm·min^−1^. A minimum of six samples were tested for each type of composite. A Hirox reflected light microscope (HIROX-USA, inc., Hackensack, NJ, USA) was used to image the fracture region of the samples, post mechanical tests.

## 3. Results and Discussion

**Figure 1:**
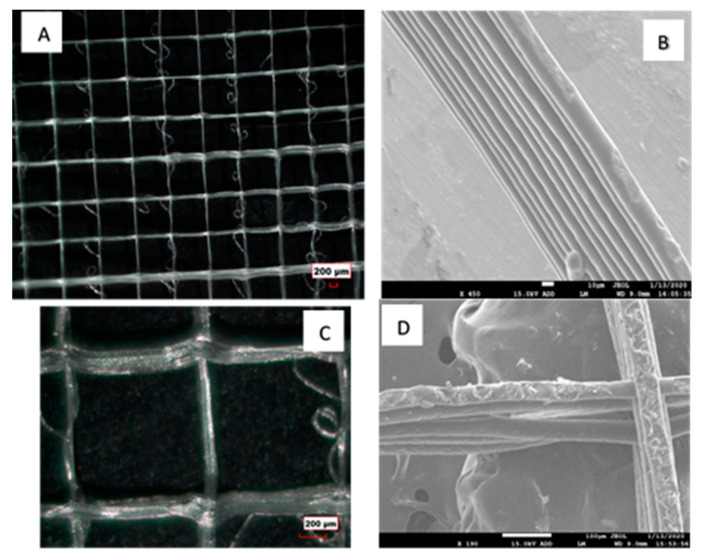
(**A**) Optical microscope image of the polycaprolactone (PCL) fibers printed as grid structures using melt electrospinning writing method; (**B**) scanning electron microscope (SEM) image of the cross-section of the grid structure (scale bar 10 µm). The fibers are deposited on top of each other in a controlled manner; (**C**) optical microscope image of the PCL fibers demonstrating the cross-over points of the grid structures; and (**D**) SEM image demonstrating the surface morphology of the fibers (scale bar 100 µm).

The morphology and size of the fibers obtained using melt electrospinning writing can be greatly influenced by the processing parameters, such as the melt temperature, distance between the nozzle and the collector, voltage used, and the X-Y velocity of the extruder head [27,28]. In our study, we used 3 kV as the melt electrospinning voltage and a 5 mm distance between the nozzle and the collector. The use of a low voltage ensured that the polymer jet ejected from the nozzle did not experience the whipping instabilities. The polymer jet travelled in a straight path towards the collector, which enabled us to deposit fibers in a controllable fashion. Figure 1A shows the optical microscope image of the PCL fibers obtained using the melt electrospinning writing method. It was evident from the microstructure (Figure 1) that the fibers were deposited in a precise and controlled manner to obtain the grid pattern. Figure 1B shows the cross-sectional image of the fiber structure. The cross-sectional image was obtained by fracturing the sample under liquid nitrogen. The scanning electron microscope (SEM) image shows that fibers within a square were deposited on top of each other in a precise and controllable manner. As the fibers were melt extruded out of a nozzle, they appeared to fuse with the neighboring layer of fibers when they were deposited on top of each other. The average diameter of the fibers within this grid pattern was determined to be ~30 µm from the SEM images (Figure 1B,C). The size of the fibers can be further reduced by controlling the electrospinning parameters. The size of the fiber obtained using the melt-electrospinning writing technique was below the fiber size obtained by using traditional fused deposition modelling (FDM). The size of the fibers that are typically obtained using FDM are 100 µm or larger [29,30,31].

These cross-ply fibrous structures were then embedded within a PVA matrix to obtain PVA reinforced with PCL fibers. The use of these cross-ply fibrous structures should play a role in improving the mechanical properties of the PVA matrix. Traditionally, non-woven fiber membranes, obtained using conventional electrospinning are used to reinforce polymer matrices. The reinforcement potential of the fibers is not fully exploited when using non-woven membranes, as the fibers are not fused to one another, and hence the fibers tend to slide over one another when a load was applied. On the contrary, these cross-ply fibrous structures, obtained using melt-electrospinning writing, had interlocked fibers at the cross over points (Figure 1C). These cross-over points were shown to be structurally resistant, which plays a significant role in reinforcing the matrix [29,30,31]. Figure 1D shows the surface morphology of the fibers. The surface morphology of some of the fibers are seen to be rough. This may indicate that the polymer did not get a chance to melt completely during the extrusion process.

**Figure 2:**
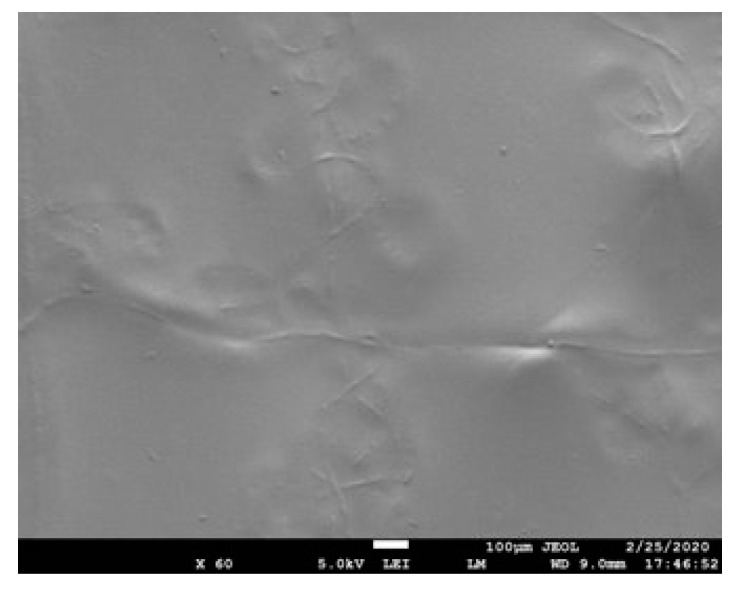
SEM image of the polyvinyl alcohol (PVA) reinforced with PCL fibers. The image shows that the PCL fibers are embedded within the PVA matrix phase.

To prepare the composite, the PVA solution was fed into a syringe, and the solution was electrosprayed on the three-dimensional PCL fiber structure at a constant rate and along the length of the fibers in both the X and Y directions. The PVA solution is seen to easily wet the PCL fibers and fill the porous regions between the fibers. The samples are then left to dry in a vacuum desiccator at room temperature. Figure 2 shows the microscope image of the composite. The SEM image shown in Figure 2 demonstrates the surface morphology of the composite. PVA appears to have penetrated the fibrous structure to form the matrix phase. It also showed that the fibers were indeed embedded within the PVA phase. The thickness of the samples was determined to be ≈120 μm. For comparison purposes, a pure PVA sample was also produced using the identical steps. The fiber to matrix ratio in the composites were maintained at 1:1 (wt/wt).

**Figure 3:**
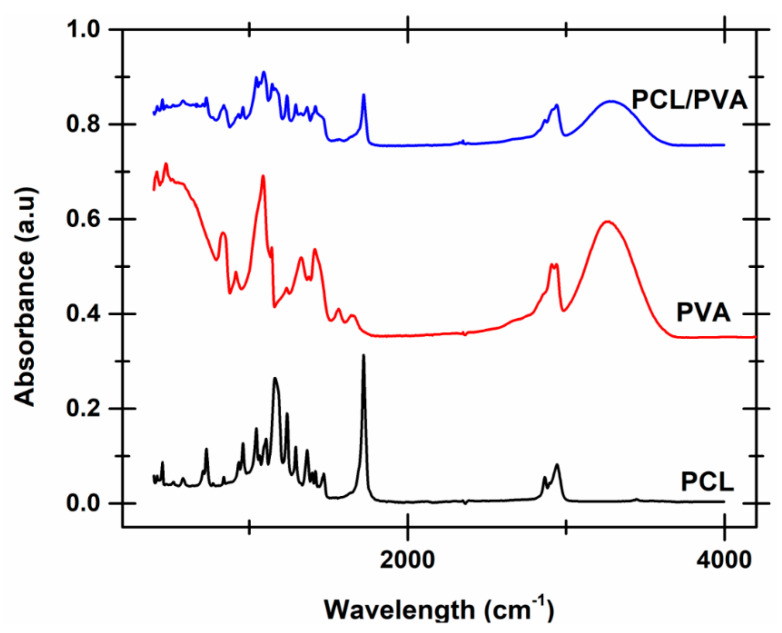
FTIR scans recorded for neat PVA and PVA reinforced with PCL fibers. The FTIR scans of neat PCL is also shown in the plot for comparison purposes.

The FTIR spectra of the PCL fibers, neat PVA, and PVA reinforced with PCL fibers are illustrated in Figure 3. FTIR spectra are also recorded to determine any chemical interaction between PCL and PVA. FTIR spectrum of the composite shows the presence of the characteristic functional groups associated with both PCL and PVA. For example, the broad peak detected at 3250 and 1410 cm^−1^ correspond to the O–H stretching and O–H bending of the hydroxyl groups of PVA [32]. The vibrational peaks seen at 2906 cm^−1^ and 2940 cm^−1^ are assigned to the asymmetric stretching of methylene group C–H_2_ of PVA. The peaks occurring between 1700 and 1640 cm^−1^ are assigned with C=C stretching and C=O groups of the PVA, while the peak between 1078 to 1300 cm^−1^ is assigned with C–O stretching [33]. These peaks are also seen in the spectra of the composite.

Similarly, all the characteristic peaks associated with PCL were also found in the PVA reinforced with PCL fibers. For example, the peaks at 2943 cm^−1^ and 2864 cm^−1^ were associated with the asymmetric and symmetric stretching vibration of CH_2_ groups of PCLs [34,35]. The characteristic peak of PCL at 1290 cm^−1^ is associated with the stretching vibration of C–C. Additionally, the peaks at 1720, 1240, and 1164 cm^−1^ correspond to C=O (carbonyl stretching), stretching vibration of C–O and stretching vibrations of C–O–C groups of PCLs. All these peaks were also evident in the FTIR spectra recorded for the composite sample. It was interesting to note that the intensity of the peaks at 3289 cm^−1^ and 2943 cm^−1^ corresponding to the –OH group and –CH– recorded in the spectra of the composite decreased in comparison to the peaks of the PVA. Similarly, the intensity of the peak at 2940 cm^−1^ in the composite corresponding to the –CH– group increased in comparison to the peak recorded in the spectra of PCL. These results indicate that there is some degree of interaction between carboxyl and hydroxyl groups of the PCL fibers and the PVA matrix [36].

**Figure 4:**
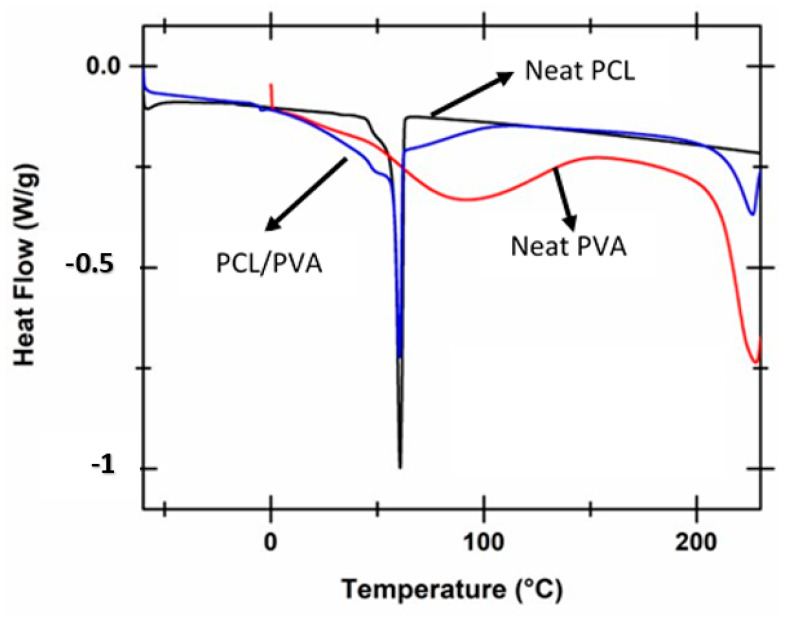
DSC scans recorded for neat PVA, neat PCL fibers, and PVA reinforced with PCL fibers.

In the next step, the thermal behavior of the samples was investigated. Figure 4 shows the DSC thermal scans recorded for neat PCL fibers, neat PVA, and PVA reinforced with PCL fibers composite. The DSC scans for neat PCL and PVA reinforced with PCL fibers displays a sharp crystalline melting peak that is associated with the melting of PCL crystals [37,38]. PVA reinforced with PCL fibers displays two melting peaks. The first melting peak seen at 60 °C is attributed to the melting of the crystals in the PCL phase, and the second peak seen at 225 °C is attributed to the melting of crystals in the PVA phase. The degree of crystallinity within the PCL phase for the samples was determined by taking the ratio between the experimental heat of fusion determined for the samples and theoretical heat of fusion for the pure crystal (136 J/g) [39]. The degree of crystallinity of the neat PCL fiber sample was determined to be 34%, while the degree of crystallinity within the PCL phase in the PVA reinforced with PCL fibers was determined to be 30%. A moderate decrease in the degree of crystallinity in the PCL phase was recorded. However, it was evident that the melting peak in neat PCL fibers was seen to be sharper and longer. On the other hand, the melting peak of PCL in the composite was wider and shallower. This may be attributed to the loss of moisture from the hydrophilic PVA within the composite.

**Figure 5:**
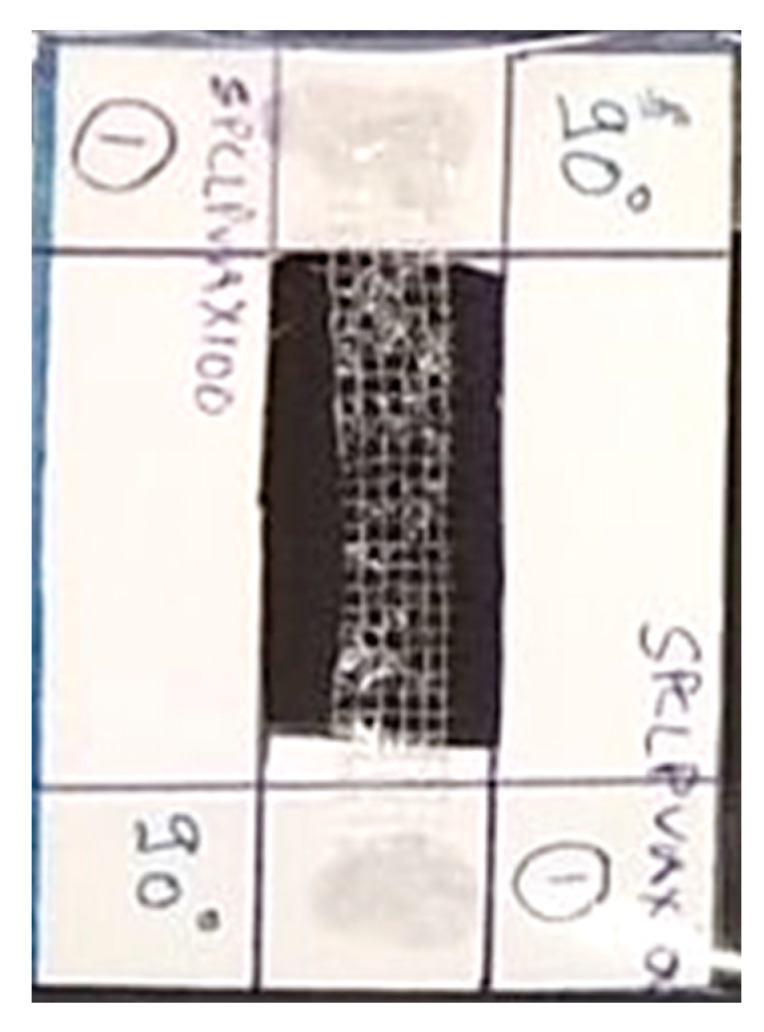
Representative digital photograph of the sample mounted on a trimmed cardboard sheet. The sample was glued firmly to the cardboard before it was inserted within the grips of a tensile tester. Once the sample and ends of the cardboard sheet were between the grips of a tensile tester, the cardboard sheet was cut to ensure that the load was experienced only by the sample.

Following this, the mechanical deformation behavior of neat PVA and PVA reinforced with PCL fibers was investigated. For this purpose, neat PVA samples and PVA reinforced with PCL fibers samples of known dimensions were taken and mounted on a trimmed cardboard sheet, as shown in Figure 5. The sample and the cardboard sheet were fixed between the grips of a tensile tester, and the cardboard sheet was cut so that the load was experienced by the sample.

**Figure 6:**
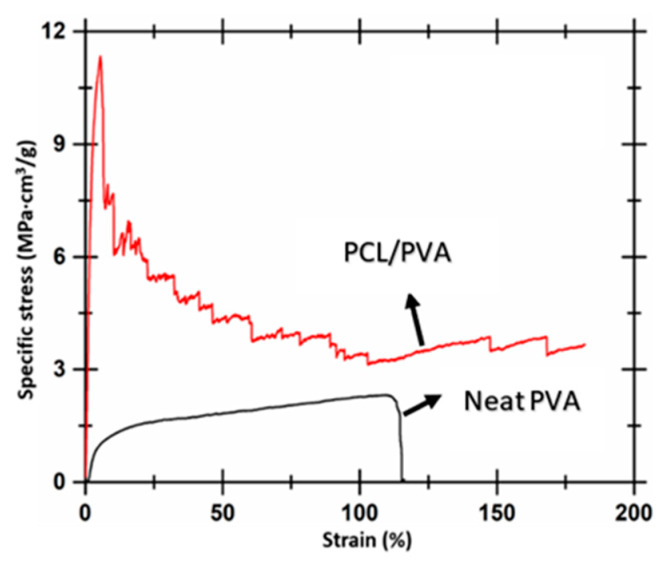
Representative specific stress versus strain curves recorded for neat PVA and PVA reinforced with PCL fibers.

The mechanical deformation behavior was evaluated from the tensile test, and the stress versus strain for both the samples were determined. Average porosity within the composite was measured using the standard porosity measurement function of Image J. Porosity within the composite was measured from three SEM images, taken from different regions of the sample. The porosity of the composite samples was determined to be 5.05% and subtracted from the original area to determine the engineering stress and strain. The Young’s modulus of neat PVA and PVA reinforced with PCL fibers was determined from the engineering stress versus strain data to be 0.23 MPa and 0.3 MPa, respectively. The improvement in the modulus can be attributed to the presence of the PCL fibers within the matrix. However, only a moderate improvement in modulus value was recorded. The moderate improvement in the modulus can be due to the decrease in crystallinity % in the PCL phase present within the composite. To get a better understanding of the reinforcing effect of the PCL fibers within the composite, the density of the matrix within the composite was determined. The density of the matrix within the composite was not considered while determining the engineering stress. A similar approach for determining the mechanical properties of polymer derived textile fabrics and fabric composites was reported in literature [40,41,42]. These studies determined the density of the composite materials to obtain the strength to weight ratio. This approach enabled them to determine the specific stress experienced by the composite during the tensile tests. The density of the matrix within the composite was determined from the ratio between the mass of the PVA within the composite to the volume of the composite. The density of the matrix was determined to be 0.084 g/cm^3^. Specific stress as a function of strain was then determined for both the samples. Specific stress experienced by the samples was determined by taking the ratio between the engineering stress and density of the matrix material within the sample. Figure 6 shows the representative specific stress versus strain curves for neat PVA and PVA reinforced with PCL fibers. Average specific strength for PVA and PVA reinforced with PCL fibers was determined to be 2.23 ± 0.21 MPa·cm^3^/g and 11.34 ± 0.51 MPa·cm^3^/g, respectively.

The toughness of the sample was determined from the area under the specific stress vs. strain curves. A similar approach was used in literature to determine the specific toughness of fiber reinforced composites [43,44]. Average specific toughness of PVA and PVA reinforced with PCL fibers were determined to be 2.05 ± 0.75 J/g and 7.81 ± 1.81 J/g, respectively. It was evident that the composite sample was stronger and tougher than the bulk matrix (PVA). This can be attributed to the interaction between the carbonyl and hydroxyl groups of PCL and PVA phase, as determined by the FTIR. The interaction between these groups was due to van der Waals interaction or hydrogen bonding between the carbonyl and hydroxyl groups. The composite sample demonstrates the ability to bear higher loads, by allowing larger strain to occur. The load in the composite fibers was shared by both the fibers and matrix. The composite samples absorb a higher amount of energy in their effort to pull the fibers in the direction of the applied load. The strain at break recorded for the composite sample was determined to be ≈ 0.4 times higher than the strain at break displayed by the PVA sample. Blond et al. [44] have reported similar specific toughness values for a PVA matrix obtained using solution casting technique. They found the specific toughness of PVA to be 2.5 J/g. In the next step, they prepared PVA solution dispersed with 0.43 vol % carbon nanotubes and electrospun this solution to obtain a PVA membrane reinforced with nanotubes. The specific toughness of these composites was determined to be 11 J/g. We showed the specific toughness of PVA composite reinforced with PCL fibers to be 7.81 J/g. The toughness can be further increased if the interfacial interaction between the matrix and the fiber phases is improved. These results show that fibers obtained using melt-electrospinning writing can be used for reinforcement purposes.

## 4. Conclusions

The results presented in this manuscript demonstrate the potential of using the fibers obtained from melt-electrospinning writing for composite reinforcement. The mechanical properties of the composites can be further improved, by improving the interfacial interaction between the fibers and the matrix. Reducing the fiber size and controlling the fiber alignment within the composite is also expected to play a significant role in influencing the mechanical properties of the composite. The results demonstrate that the inclusion of PCL fibers within the PVA matrix improves the mechanical properties of the PVA composite. The specific strength of the composite matrix was improved by five times, compared to the specific strength of the neat PVA film. This is attributed to the presence of PCL fibers within the matrix, and the interaction between the PCL fibrous phase and the PVA matrix phase within the composite. Our future studies will explore the effect of fiber size on the mechanical properties of the composite.
